# Imaging CXCL12-CXCR4 Signaling in Ovarian Cancer Therapy

**DOI:** 10.1371/journal.pone.0051500

**Published:** 2013-01-23

**Authors:** Emma Salomonnson, Amanda C. Stacer, Anna Ehrlich, Kathryn E. Luker, Gary D. Luker

**Affiliations:** 1 Center for Molecular Imaging, Department of Radiology, University of Michigan Medical School, Ann Arbor, Michigan, United States of America; 2 Department of Microbiology and Immunology, University of Michigan Medical School, Ann Arbor, Michigan, United States of America; 3 Department of Biomedical Engineering, University of Michigan Medical School, Ann Arbor, Michigan, United States of America; Baylor College of Medicine, United States of America

## Abstract

Chemokine CXCL12 and receptor CXCR4 have emerged as promising therapeutic targets for ovarian cancer, a disease that continues to have a dismal prognosis. CXCL12-CXCR4 signaling drives proliferation, survival, and invasion of ovarian cancer cells, leading to tumor growth and metastasis. Pleiotropic effects of CXCR4 in multiple key steps in ovarian cancer suggest that blocking this pathway will improve outcomes for patients with this disease. To quantify CXCL12-CXCR4 signaling in cell-based assays and living mouse models of ovarian cancer, we developed a click beetle red luciferase complementation reporter that detects activation of CXCR4 based on recruitment of the cytosolic adapter protein β-arrestin 2. Both in two-dimensional and three-dimensional cell cultures, we established that bioluminescence from this reporter measures CXCL12-dependent activation of CXCR4 and inhibition of this pathway with AMD3100, a clinically-approved small molecule that blocks CXCL12-CXCR4 binding. We used this imaging system to quantify CXCL12-CXCR4 signaling in a mouse model of metastatic ovarian cancer and showed that treatment with AMD3100 interrupted this pathway *in vivo*. Combination therapy with AMD3100 and cisplatin significantly decreased tumor burden in mice, although differences in overall survival were not significantly greater than treatment with either agent as monotherapy. These studies establish a molecular imaging reporter system for analyzing CXCL12-CXCR4 signaling in ovarian cancer, which can be used to investigate biology and therapeutic targeting of this pathway in cell-based assays and living mice.

## Introduction

Chemokine CXCL12 (SDF-1) and its receptor CXCR4 are strongly implicated as key determinants of tumor initiation and intraperitoneal metastasis of ovarian cancer [1]. CXCL12 is secreted by ≈70–90% of ovarian cancer cells, as well as mesothelial cells within the peritoneum of humans and mice [2] [3] [4] [5]. Patients with the highest levels of CXCL12 expression in ovarian cancer cells have a significantly worse prognosis, emphasizing the biologic significance of this signaling molecule in disease progression [6]. Effects of CXCL12 on ovarian cancer appear to be mediated by CXCR4, one of two known receptors for this chemokine. Amplification of CXCR4 is an early event in malignant transformation of ovarian epithelial cells, suggesting that CXCL12-CXCR4 is critical for pathogenesis of this disease [7]. Approximately 60% of patients with ovarian cancer have CXCR4 on malignant cells, and these patients have significantly reduced overall survival [4]. CXCL12 signaling through CXCR4 promotes proliferation, invasion, and metastasis of ovarian cancer cells, all of which contribute to more aggressive disease. Adverse effects of CXCL12 on ovarian cancer also may be due to effects on the stromal compartment of the tumor microenvironment, including enhanced angiogenesis and recruitment of immunosuppressive cells [8] [9] [10] [11].

Central functions of CXCL12-CXCR4 signaling in malignant cells and the tumor microenvironment position this signaling axis as a key target to improve therapy for patients with ovarian cancer. In mouse models, we and others have shown that blocking CXCL12-CXCR4 signaling with RNA interference against CXCL12 or a small molecule inhibitor of CXCL12-CXCR4 binding (AMD3100) limits growth of ovarian cancer cell implants [12], [13], [14]. Inhibiting CXCL12-CXCR4 signaling also extends survival of mice with ovarian cancer. However, effects of single agent therapy are modest, suggesting that targeting CXCL12-CXCR4 in combination with another agent may be more beneficial.

Establishing that a compound effectively hits its intended target is essential for drug development in pre-clinical models and clinical trials. To analyze pharmacodynamics of agents targeting CXCL12-CXCR4 signaling in mouse models of ovarian cancer, we developed a click beetle luciferase complementation reporter to image and quantify activation and inhibition of this pathway *in vivo*. In this system, CXCR4 and the cytosolic adapter protein β-arrestin 2 are fused to inactive amino (CBRN) and carboxy (CBC) terminal fragments of click beetle red luciferase. The reporter measures CXCL12 activation of CXCR4 signaling based on recruitment of the cytosolic adapter protein β-arrestin 2 to this receptor. Recruitment of β-arrestin 2 is a common, early event in activation of chemokine receptors and the larger family of seven transmembrane receptors [15]. With the luciferase complementation system, interactions between CXCR4 and β-arrestin 2 reconstitute active click beetle luciferase to produce light, providing an imaging metric for CXCR4 signaling in intact cells, three-dimensional spheroid cultures, and living mice. Spheroids are an important intermediate between standard two-dimensional cell culture assays and tumor xenografts since tumor spheroids reproduce restricted diffusion of compounds in tumors and formation of ovarian cancer spheroids in ascitic fluid from patients. Since luciferase complementation is reversible, this imaging reporter also can be used to measure effects of compounds blocking CXCL12-CXCR4 signaling *in vitro* and *in vivo*.

We used this imaging reporter to analyze CXCL12-CXCR4 signaling in ovarian cancer and establish that AMD3100, a small molecule inhibitor of CXCR4, blocks receptor activation in the tumor microenvironment. Using this imaging system, we determined that combination therapy with AMD3100 and cisplatin significantly decreased overall tumor burden in a mouse model of human ovarian cancer with intraperitoneal metastases. These results establish a new molecular imaging method to analyze CXCL12-CXCR4 signaling in ovarian cancer and suggest possible therapeutic benefit of combining selective inhibition of this chemokine receptor pathway with standard chemotherapeutic drugs.

## Materials and Methods

### Plasmids

**Figure 1:**
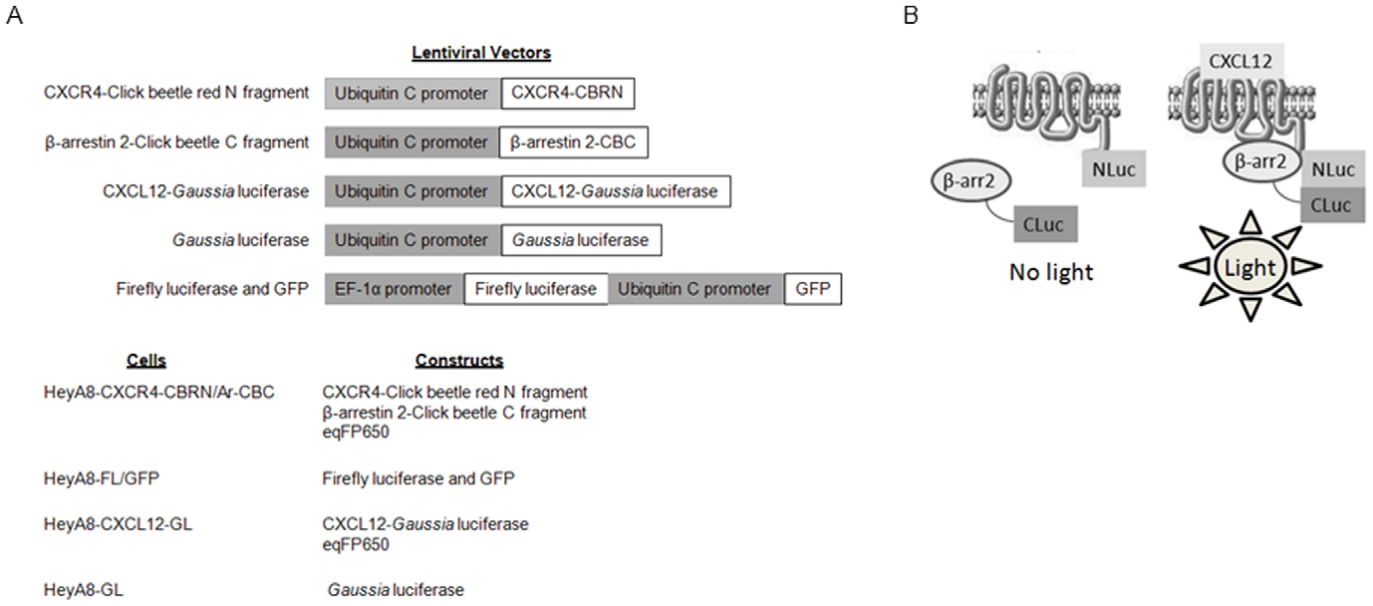
Image reporters, transduced cells, and click beetle complementation system. A) Panel shows lentiviral vectors and stably transduced cells used for imaging studies. B) Schematic diagram of click beetle red complementation system. N- and C-terminal fragments of click beetle red luciferase are fused to the C-termini of CXCR4 and β-arrestin 2, respectively. CXCL12 binding to CXCR4 causes recruitment of β-arrestin 2 to the activated receptor, reconstituting click beetle red luciferase to produce light.

To generate fusions of CXCR4 with the N-terminal fragment of click beetle red luciferase (CBRN) (Promega), we amplified CXCR4 by PCR and cloned the product into the XhoI and AgeI sites of EGFP-N1 (Clontech). We used PCR to amplify the DNA sequence for amino acids 2–413 of click beetle red luciferase (CBRN) and cloned this product into AgeI and NotI sites of EGFP-N1 [16]. This cloning strategy removes EGFP from the vector. We amplified the sequence for amino acids 395–542 of click beetle luciferase (CBC) by PCR and cloned the product into the AgeI and NotI sites of EGFP-N1 to create a fusion to the C-terminus of β-arrestin 2 (gift of Robert Lefkowitz). To transfer constructs from the EGFP-N1 backbone to lentiviral vector FUW, we used PCR to amplify the target DNA sequence and add XbaI sites for cloning. Constructs used for this study are summarized in Figure 1 (Fig. 1A), and PCR primers used for cloning are listed in Table 1.

**Table 1:** PCR primers.

Target	Primer
CXCR4	5′- atgcctcgaggccaccatggaggggatcagtatatacacttc-3′
	5′-gcataccggtgcgctggagtgaaaacttgaagactc-3′
Click beetle N-terminal fragment (CBRN)	5′-atgcaccggtcggcggtggctcatctggcggaggtgtaaagcgtgagaaaaatgtcatctatg-3′
	5′- gcatgcggccgcttagccgtcgtcgtcgatggcc-3′
Click beetle C-terminal fragment (CBC)	5′-atgcaccggtcggcggtggctcatctggcggaggtagcaagggttatgtcaataacg-3′
	5′-gcatgcggccgcttaaccgccggccttctccaacaattgtttc-3′
Transfer to FUW lentiviral vector	5′-cgattctagagctaccggactcagatctcgag-3′
	5′- atgtggtatggctgattatgatc-3′

### Cells

HeyA8 ovarian cancer cells (provided by Gordon Mills, MD Anderson Cancer Center) were stably transduced with recombinant lentiviruses for CXCR4-CBRN and Ar-CBC (HeyA8-CXCR4-CBRN/Ar-CBC) [17]. We used lentiviral transduction to establish populations of HeyA8 cells stably expressing CXCL12 fused to *Gaussia* luciferase (HeyA8-CXCL12-GL), unfused *Gaussia* luciferase (Hey-GL), and firefly luciferase and green fluorescent protein (GFP) (HeyA8-FL/GFP) [18], [19]. We also transduced HeyA8-CXCR4-CBRN/Ar-CBC and HeyA8-CXCL12-GL cells with fluorescent protein eqFP650 [20]. Figure 1 shows a list of stably transduced cells used in this study (Fig. 1A). All cells were maintained in DMEM with 10% fetal bovine serum, 1% glutamine, and 0.1% penicillin/streptomycin.

### Flow cytometry

Intact cells were stained with an antibody to CXCR4 (clone 12G5, R&D Systems) or matched isotype control as described previously to reveal cell surface levels of this receptor [21]. Unstained cells from each cell population were used as compensation controls for flow cytometry.

### Western blotting

Cells were cultured in medium containing 1% serum overnight and then stimulated for 10 minutes with various concentrations of recombinant CXCL12-α (R&D Systems) for activation of AKT. To determine CXCL12-dependent activation of ERK1/2, we treated serum-starved cells with 100 ng/ml CXCL12-α for 0, 5, 15, or 30 minutes in the presence of vehicle control or 1 µM AMD3100, a small molecule inhibitor of CXCR4 (Tocris). Cell lysates were blotted for phosphorylated AKT or phosphorylated ERK1/2 (Cell Signaling) as described previously [19]. Blots were stripped and re-probed for total AKT or total ERK1/2 as a loading control. We also used Western blotting to determine expression of endogenous β-arrestin 2 and β-arrestin 2-CBC in HeyA8-CXCR4-CBRN/Ar-CBC cells (Cell Signaling). Membranes were stripped an additional time and re-probed for glyceraldehyde phosphate dehydrogenase (GAPDH) as a further loading control. We measured intensities of bands with ImageJ and divided values for phosphorylated AKT by total AKT and GAPDH. We performed similar calculations for phosphorylated ERK relative to total ERK and GAPDH. For both phosphorylated AKT and ERK, we normalized all values to cells not treated with CXCL12.

### Two dimensional cell culture experiments

We plated 1.5×10^4^ HeyA8-CXCR4-CBRN/Ar-CBC cells per well in black wall 96 well plates one day before assays. We changed medium from standard culture medium to DMEM with 1% serum for experiments. We incubated cells for increasing periods of time with 100 ng/ml CXCL12-α (R&D Systems) or for 60 minutes with increasing concentrations of chemokine. In selected experiments, cells were incubated with AMD3100 to inhibit CXCL12 binding to CXCR4 or vehicle control at concentrations listed in figure legends. We added 150 µg/ml luciferin (Promega) to wells and then quantified bioluminescence from living cells using an IVIS 100 (Perkin Elmer). Bioluminescence from click beetle luciferase was quantified as described previously and normalized to total protein per well quantified by sulforhodamine B staining [19]. Data were expressed as mean values ± SEM for luminescence relative to untreated controls.

### Spheroids

Spheroids were formed in 384-well hanging drop plates by seeding a total of 2×10^4^ HeyA8 cells per well [22]. We used two different types of spheroids: 1) HeyA8-CXCR4-CBRN/Ar-CBC cells mixed with equal numbers of HeyA8-CXCL12-GL cells to assay activation and inhibition of CXCR4 signaling; or 2) HeyA8-FL/GFP cells combined with equal numbers of either HeyA8-CXCL12-GL or HeyA8-GL cells, respectively, for assays of cell viability in response to cisplatin. For experiments with AMD3100, increasing concentrations of compound were added to spheroids one day after seeding in hanging drop plates. We incubated spheroids with AMD3100 or vehicle for one or two days before quantifying bioluminescence. HeyA8-FL/GFP cells in spheroids with either HeyA8-CXCL12-GL or HeyA8-GL cells were treated for one or two days with increasing concentrations of cisplatin. We quantified fluorescence from eqFP650 on an IVIS Spectrum prior to quantifying bioluminescence after adding 6 µg/ml luciferin to each spheroid. We normalized bioluminescence photon flux to fluorescence radiance to account for differences in cell numbers.

### Animal studies

All animal procedures were approved by the University of Michigan Committee for the Use and Care of Animals. Animals were switched to chlorophyll-free chow (Research Diets) for all studies to minimize background fluorescence in imaging studies. 2.5×10^5^ cells each of HeyA8-CXCR4-CBRN/Ar-CBC and HeyA8-CXCL12-GL cells were injected intraperitoneally in 100 µl 0.9% NaCl into 5–7 week old female NOD/SCID *IL2rγ* ^−/−^ female mice (Taconic). To inhibit CXCL12 binding to CXCR4, we used 5-day, 1.0 µl/hour osmotic pumps (for the experiment shown in Fig. 5) or 14-day, 0.5 µl/hour osmotic pumps (for the experiment shown in Fig. 6) (Alzet) loaded with 25 mg/ml AMD3100 or 0.9% NaCl vehicle control. These pumps deliver 1.25 or 0.625 µg AMD3100/g/hour to each mouse. Pumps were implanted at times indicated in the text for each figure. For treatment studies shown in Figure 6, we injected mice with 4 mg/kg cisplatin or matched vehicle i.p. every 5 days. Cisplatin or PBS vehicle injections continued throughout the two-week period that osmotic infusion pumps were in place. All mice received active compound (AMD3100 and/or cisplatin) or matched vehicle for both routes of delivery (osmotic infusion pump and i.p. injection). As examples, vehicle control mice received osmotic pumps with 0.9% NaCl and i.p. injections of PBS, while mice in the AMD3100 treatment group had osmotic pumps with AMD3100 and i.p. PBS.

### Mouse imaging

Bioluminescence imaging was performed on an IVIS Spectrum (Perkin Elmer). Fluorescence imaging and beetle luciferase imaging with luciferin were performed as described previously [23]. Imaging data were quantified as fluorescence radiance or photon flux, respectively. Data for click beetle red luciferase complementation were normalized to total tumor burden assessed by fluorescence from eqFP650.

### Statistics

Graphs and statistical analyses were prepared with GraphPad Prism. Cell culture studies were performed 3–5 times, while animal studies were performed twice. Data were plotted as mean values with standard error of the mean (SEM). Pairs of data were analyzed by Mann-Whitney U test to determine statistically significant differences. Kaplan-Meier survival curves were analyzed by Gehan-Breslow-Wilcoxon Test.

## Results

### Click beetle complementation reporter for interaction of CXCR4 and β-arrestin 2

CXCL12 binding to receptor CXCR4 results in recruitment of the cytosolic adapter protein β-arrestin 2 to the activated receptor. To image and quantify this key step in signal transduction, we used a recently described protein fragment complementation assay based on click beetle red luciferase [16]. In this system, CXCR4 is fused to the N-terminal fragment of click beetle luciferase (CXCR4-CBRN) and β-arrestin 2 to the C-terminal fragment of this enzyme (Ar-CBC) (Fig. 1B). Recruitment of β-arrestin 2 to CXCR4 also brings together luciferase fragments to produce bioluminescence as a quantitative measure of this protein interaction in CXCR4 signaling.

**Figure 2:**
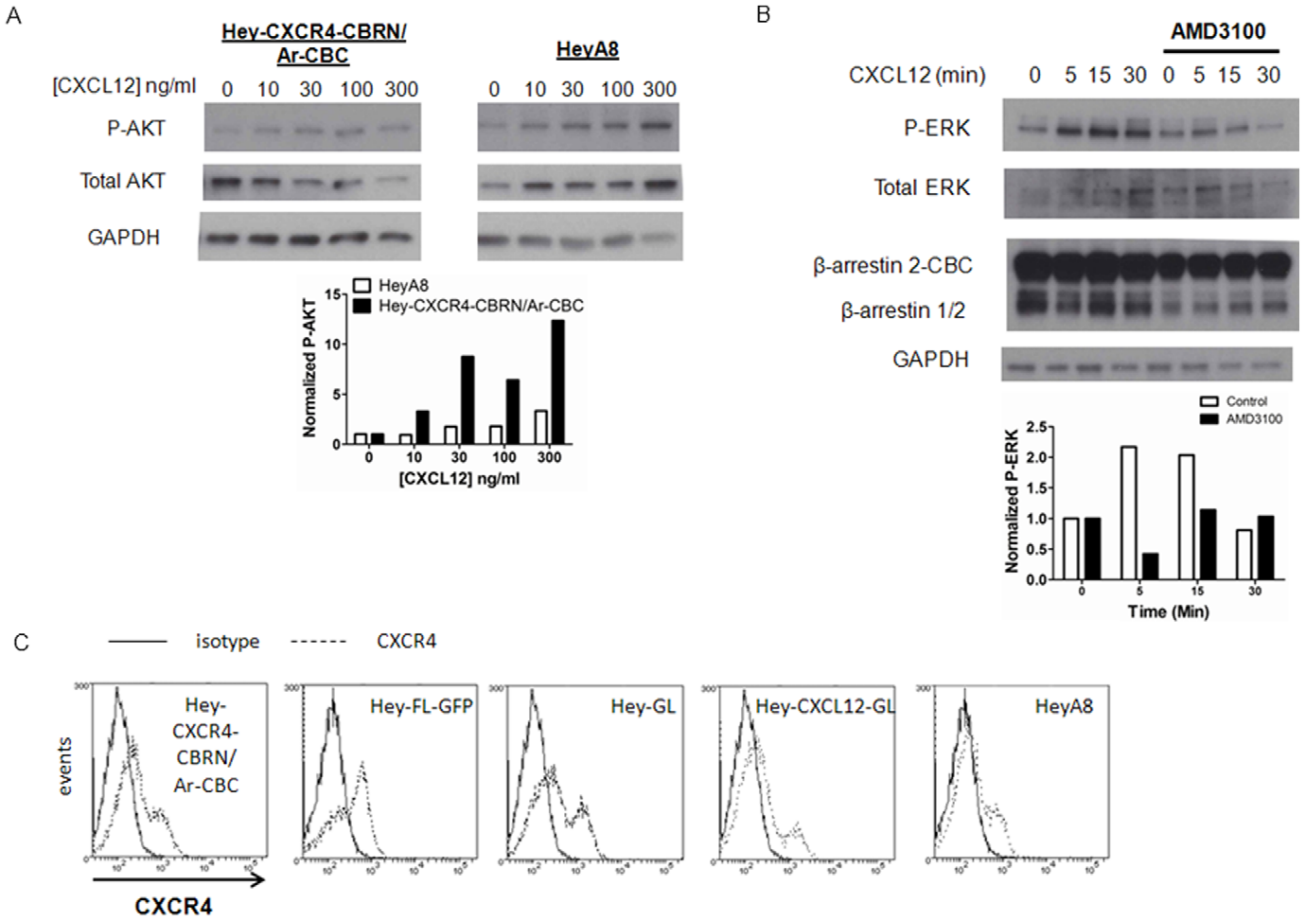
CXCL12-CXCR4 signaling pathway in ovarian cancer cells. A) HeyA8-CXCR4-CBRN/Ar-CBC and parental HeyA8 cells were treated with increasing concentrations of CXCL12 for 10 minutes. Western blot of total cell lysates shows phosphorylated and total AKT, respectively. We used GAPDH as a loading control. Graph shows relative band intensities for phosphorylated AKT in each cell line normalized to total AKT and GAPDH. B) HeyA8-CXCR4-CBRN/Ar-CBC cells were treated with 100 ng/ml CXCL12-α for 0, 5, 15, or 30 minutes in the absence or presence of 1 µM AMD3100. Total cell lysates were probed for phosphorylated and total ERK1/2, respectively. Lysates also were analyzed by Western blot for expression of β-arrestin 2-CBC and endogenous β-arrestin 1 and 2. GAPDH is shown as a loading control. C) Flow cytometry of CXCR4 expression in various HeyA8 cell lines used in this study. Dark line and dashed lines in histogram plots denote isotype control and staining with CXCR4 antibody.

We transduced HeyA8 ovarian cancer cells with lentiviral vectors for CXCR4-CBRN and Ar-CBC. HeyA8-CXCR4-CBRN/Ar-CBC cells phosphorylated AKT in response to incubation with CXCL12 as determined by Western blotting, showing that these cells activated a known downstream effector of CXCL12-CXCR4 (Fig. 2A). As compared with parental HeyA8 cells, HeyA8-CXCR4-CBRN/Ar-CBC cells showed greater activation of AKT in response to CXCL12, consistent with transducing additional functional CXCR4 into the imaging reporter cells. We also demonstrated that HeyA8-CXCR4-CBRN/Ar-CBC cells show time-dependent activation of kinases ERK1/2, which was inhibited by a saturating (1 µM) concentration of the CXCR4 inhibitor AMD3100 (Fig. 2B). HeyA8-CXCR4-CBRN/Ar-CBC cells overexpress β-arrestin 2-CBC relative to the endogenous protein as determined by Western blotting.

We used flow cytometry to assess cell surface expression of CXCR4 on HeyA8-CXCR4-CBRN/Ar-CBC cells, as well as parental HeyA8 cells and cells stably transduced with *Gaussia* luciferase (HeyA8-GL), CXCL12 fused to Gaussia luciferase (HeyA8-CXCL12-GL), or firefly luciferase and GFP (HeyA8-FL-GFP). All cells expressed similar levels of cell surface CXCR4 (Fig. 2C). Although transduced with CXCR4-CBRN, HeyA8-CXCR4-CBRN/Ar-CBC cells had levels of cell surface CXCR4 that were comparable to parental HeyA8 cells, likely because overexpression of β-arrestin 2 causes internalization of this receptor from the cell surface. [24], [25].

### CXCL12 drives complementation between CXCR4 and β-arrestin 2

**Figure 3:**
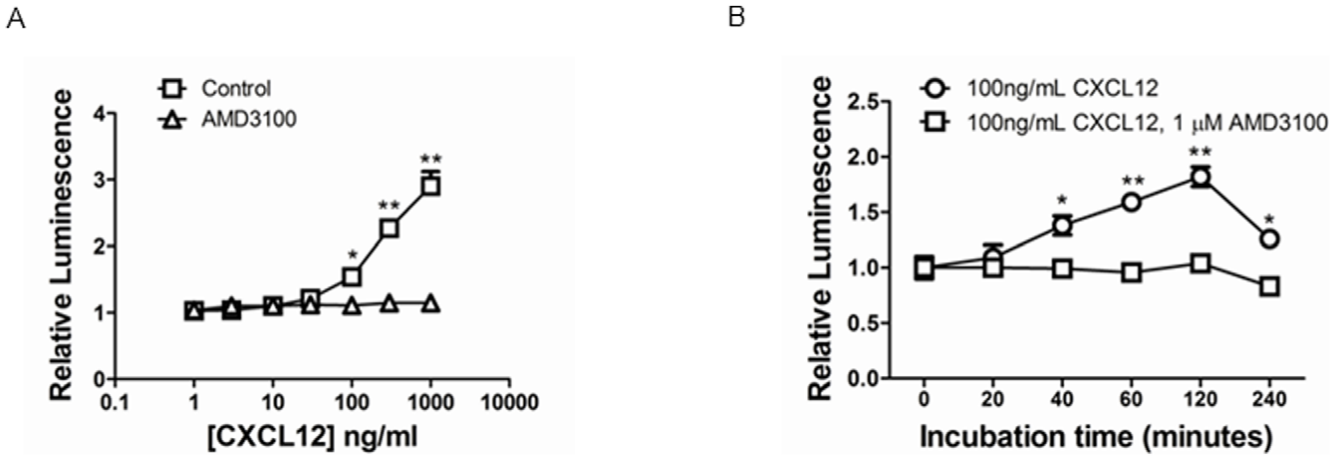
HeyA8-CXCR4-CBRN/Ar-CBC cells report on receptor activation. A) HeyA8-CXCR4-CBRN/Ar-CBC cells were plated as two-dimensional cultures in 96 well plates and incubated with increasing concentrations of CXCL12-α in the presence of 1 µM AMD3100 or vehicle control. Data were graphed as mean values for luminescence relative to cells not treated with CXCL12 (n = 4 per condition). Error bars denote SEM. B) HeyA8-CXCR4-CBRN/Ar-CBC cells were treated with 100 ng/ml CXCL12 for increasing periods of time in the presence of 1 µM AMD3100 or vehicle control. Data were graphed as mean values ± SEM for luminescence relative to cells not incubated with CXCL12 (n = 4 per condition). *, p<0.05; **, p<0.01.

To establish that bioluminescence from HeyA8-CXCR4-CBRN/Ar-CBC cells measures activation of CXCR4 signaling, we treated monolayer cultures of these cells with increasing concentrations of CXCL12 for 60 minutes. We treated parallel cultures of cells with an inhibitory concentration (1 µM) of the CXCR4 inhibitor AMD3100 or vehicle control during the incubation period [26] [27]. Treatment with CXCL12 produced a concentration-dependent increase in bioluminescence from association of CXCR4-CBRN and Ar-CBC, reaching peak 3-fold induction at 1 µg/ml CXCL12 (Fig. 3A). By comparison, cells treated with AMD3100 showed only basal bioluminescence with no response to CXCL12.

We also incubated HeyA8-CXCR4-CBRN/Ar-CBC cells with 100 ng/ml CXCL12 and 1 µM AMD3100 or vehicle control for increasing periods of time through 4 hours before measuring luciferase activity. Relative to untreated cells, cells incubated with 100 ng/ml CXCL12 and vehicle control showed time-dependent increases in bioluminescence, peaking at approximately 2-fold induction at 2 hours and then declining modestly (Fig. 3B). AMD3100 completely blocked effects of CXCL12 on bioluminescence complementation between CXCR4-CBRN and Ar-CBC. Collectively, these studies show that bioluminescence from HeyA8-CXCR4-CBRN/Ar-CBC cells responds to CXCL12 and establish that this reporter system can quantify inhibition of CXCL12-CXCR4 signaling.

### Drug effects in spheroid culture

**Figure 4:**
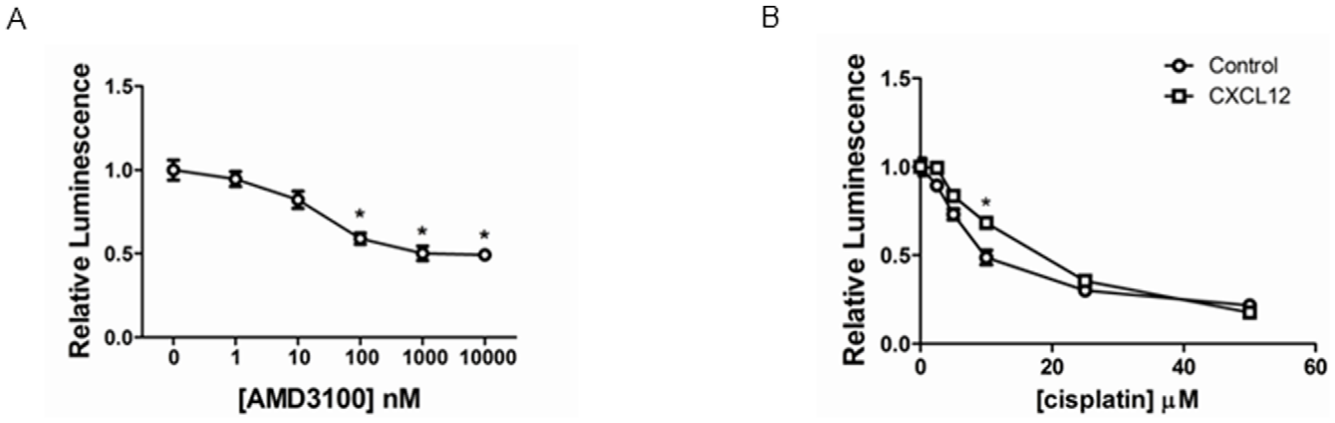
CXCR4 signaling and cell viability in spheroid cultures. A) Spheroid cultures of HeyA8-CXCR4-CBRN/Ar-CBC and HeyA8-CXCL12-GL cells were treated for 24 hours with increasing concentrations of AMD3100. Data were graphed as mean values ± SEM for luminescence for click beetle complementation relative to spheroids treated only with vehicle control (n = 10 spheroids per condition). B) HeyA8-FL-GFP cells were cultured as spheroids with HeyA8-CXCL12-GL or HeyA8-GL cells and then treated with increasing concentrations of cisplatin for 24 hours. Data were graphed as mean values ± SEM for firefly luciferase luminescence relative to spheroids not treated with cisplatin (n = 10 spheroids per condition). *, p<0.05.

Recent studies suggest that spheroids and similar three-dimensional cell culture systems are more physiologic models of drug targeting and efficacy, due to factors including direct intercellular interactions and restricted diffusion of compounds [28], [29]. In addition, ovarian cancer cells in patients form spheroids in ascites, posing a potential barrier to drug delivery [30]. To model the tumor microenvironment of ovarian cancer *in vivo*, we used spheroids of HeyA8-CXCR4-CBRN/Ar-CBC cells combined with equal numbers of HeyA8-CXCL12-GL cells, reproducing human ovarian cancer in which tumor cells secrete CXCL12 and/or express CXCR4. While parental HeyA8 cells do not express CXCL12 as determined by QRT-PCR (data not shown), HeyA8-CXCL12-GL cells secrete approximately 12 ng/ml CXCL12 in a 24 hour period, which is comparable to values reported for other ovarian cancer cells that secrete this chemokine endogenously [19] [31]. We treated spheroids with increasing concentrations of AMD3100 for 24 hours before quantifying bioluminescence. Relative to spheroids treated with vehicle control, AMD3100 inhibited bioluminescence from association of CXCR4-CBRN and Ar-CBC. Luciferase activity from the complementation reporter decreased by ≈50% in spheroids treated with 1 µM AMD3100 (p<0.05) (Fig. 4A). We observed similar results when we extended incubations to two days with AMD3100 (data not shown). Inhibition of CXCR4, as quantified by the complementation assay, was less effective in spheroids as compared with monolayer culture, suggesting that three-dimensional architecture limits penetration of AMD3100 to all cells. However, we note that spheroid cultures test effects of chronic exposure to CXCL12 rather than acute addition of chemokine as done in two-dimensional culture, which also may contribute to differences in efficacy of AMD3100.

We tested protective effects of CXCL12 against cytotoxicity from cisplatin, a standard chemotherapeutic drug for ovarian cancer. For these assays, we used HeyA8-FL-GFP cells, which endogenously express CXCR4 (see Fig. 2C). Firefly luciferase activity is directly proportional to numbers of tumor cells, providing a facile assay for cell viability in intact spheroids [32]. We generated spheroids combining HeyA8-FL-GFP cells with HeyA8-CXCL12-GL or HeyA8-GL cells, respectively, and then treated spheroids with increasing concentrations of cisplatin for 24 hours. In spheroids containing HeyA8-CXCL12-GL cells, HeyA8-FL-GFP cells were protected modestly against cytotoxic effects of cisplatin, although only at 10 µM did we detect significant differences in cytotoxicity relative to spheroids containing HeyA8-GL cells (p<0.05) (Fig. 4B). The extent of cytotoxicity was comparable following drug treatments for 48 hours (data not shown). These studies show that CXCL12 confers very modest protection against cisplatin in spheroids comprised solely of ovarian cancer cells.

### *In vivo* imaging of CXCR4 and β-arrestin 2 complementation in ovarian cancer

**Figure 5:**
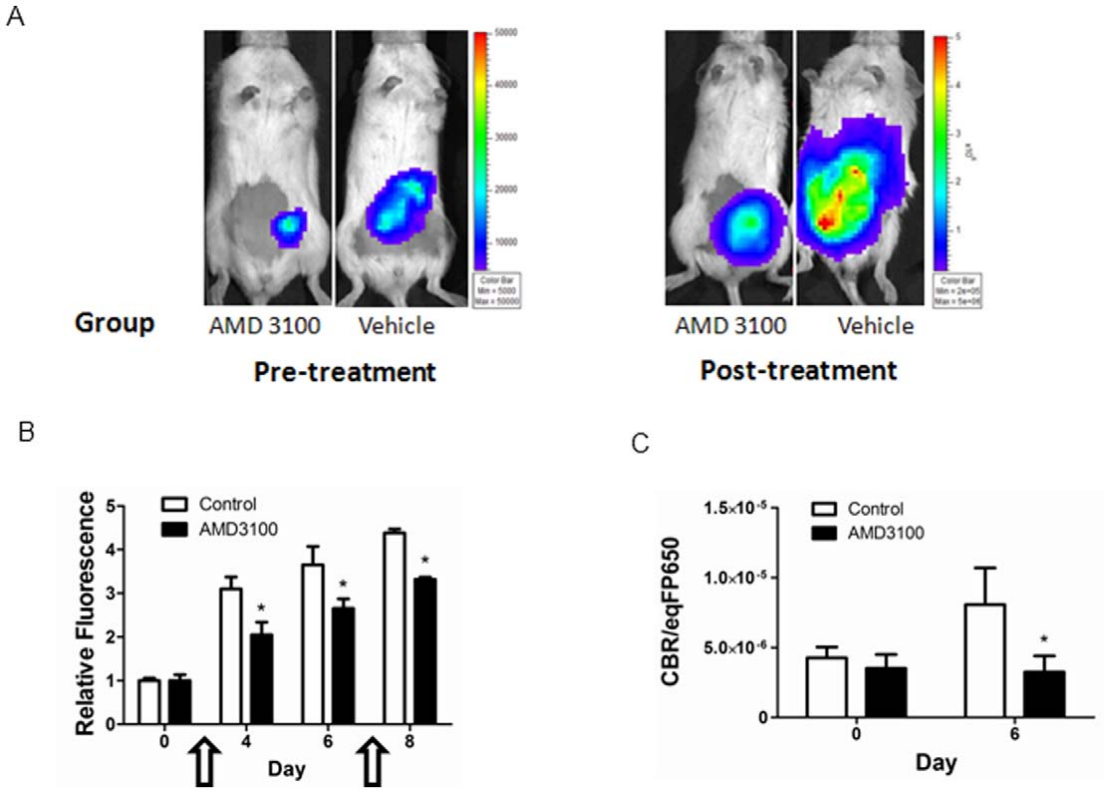
Imaging association of CXCR4 and β-arrestin 2 in living mice. A) Representative images of mice with intraperitoneal implants of HeyA8-CXCR4-CBRN/Ar-CBC and HeyA8-CXCL12-GL cells. Images were obtained before and following 5 days of treatment with osmotic pumps containing AMD3100 or vehicle control. Scale bar depicts ranges of photon flux values displayed on pseudocolor images. B) Fluorescence from ovarian cancer cells was measured in living mice treated with AMD3100 or vehicle control. Graph shows mean values+SEM for fluorescence relative to values measured on day 0 before beginning treatment. Arrows show the period when osmotic pumps were in place. C) Quantified photon flux data for click beetle red complementation in mice treated with AMD3100 or vehicle control, respectively. Data were normalized to tumor fluorescence for each mouse and graphed as mean values+SEM (n = 7 mice per group). *, p<0.05.

We used a human tumor xenograft model of metastatic intraperitoneal ovarian cancer to determine to what extent complementation between CXCR4 and β-arrestin 2 detects CXCR4 activation and inhibition *in vivo*. We co-injected equal numbers of HeyA8-CXCR4-CBRN/Ar-CBC and HeyA8-CXCL12-GL cells intraperitoneally in mice and used bioluminescence imaging to quantify recruitment of β-arrestin 2 to CXCR4. Under baseline conditions, we readily observed bioluminescence from complementation between HeyA8-CXCR4-CBRN and Ar-CBC (Fig. 5A), indicating active CXCR4 signaling in malignant cells. We then randomly separated mice into groups treated for five days with either AMD3100 or vehicle control delivered from osmotic infusion pumps. There was no phenotypic evidence of toxicity in mice treated with AMD3100. Mice treated with AMD3100 had relatively less growth of ovarian cancer cells than animals receiving only vehicle control as quantified by fluorescence from far red fluorescent protein eqFP650 expressed in malignant cells (Fig. 5B) (p<0.05). Using fluorescence from eqFP650 to normalize for differences in total numbers ovarian cancer cells, bioluminescence from interaction of CXCR4-CBRN with Ar-CBC was significantly lower in mice that received AMD3100 for five days as compared with vehicle control. Collectively, these data show the utility of this imaging system for measuring pharmacologic targeting of CXCL12-CXCR4 signaling and resultant effects of tumor growth *in vivo* (Fig. 5C) (p<0.05).

### Combination therapy with AMD3100 and cisplatin reduces tumor burden

We previously demonstrated that single agent therapy with AMD3100 alone modestly improves survival of mice with intraperitoneal disseminated ovarian cancer [14]. We hypothesized that combined therapy with AMD3100 and a standard chemotherapeutic drug would significantly improve tumor control and survival, based on studies in leukemia showing that CXCL12 in the tumor microenvironment confers resistance to standard cytotoxic drugs [33]. To test this hypothesis, we implanted mice with HeyA8-CXCR4-CBRN/Ar-CBC and HeyA8-CXCL12-GL cells. One week after implanting tumors we randomized mice to four treatment groups: 1) vehicle control; 2) AMD3100 delivered by two-week osmotic infusion pumps; 3) cisplatin administered as 4 mg/kg i.p. every 5 days; and 4) combined AMD3100 and cisplatin. We continued cisplatin injections through the two-week delivery period of AMD3100 pumps and then discontinued all therapy.

**Figure 6:**
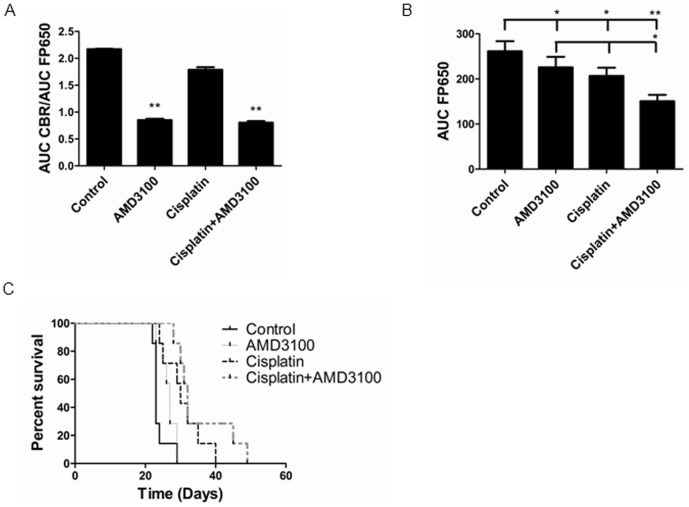
Combination therapy with AMD3100 and cisplatin decreases tumor burden. A) Area under the curve analysis of imaging data for ratios of click beetle red luciferase complementation for CXCR4 and β-arrestin 2 normalized to eqFP650 fluorescence (tumor burden) in mice implanted with HeyA8-CXCR4-CBRN/Ar-CBC and HeyA8-CXCL12-GL cells. Data are shown for groups treated with vehicle control, AMD3100, cisplatin, or both AMD3100 and cisplatin for two weeks beginning one week after implanting cells. Graph shows mean values+SEM (n = 7 mice per group). *, p<0.05. B) Area under the curve analysis of fluorescence from eqFP650 produced by implanted ovarian cancer cells over the course of the experiment. Graph shows mean values+SEM. *, p<0.05, **, p<0.01. C) Kaplan-Meier curves for survival of mice treated with vehicle, AMD3100, cisplatin, or AMD3100 and cisplatin. All treatment groups differ from vehicle control (p<0.05) but not from each other.

We used bioluminescence from HeyA8-CXCR4-CBRN/Ar-CBC to analyze CXCR4 signaling *in vivo* and fluorescence imaging for eqFP650 to quantify tumor burden over time. We calculated area under the curve for bioluminescence and fluorescence and used ratios of these parameters to assess inhibition of CXCR4 over time (Fig. 6A). Mice treated with AMD3100 or the combination of AMD3100 and cisplatin had significantly lower bioluminescence from CXCR4-CBRN and Ar-CBC complementation relative to overall tumor burden, showing that this drug successfully blocks CXCR4 signaling in ovarian cancer cells *in vivo* (p<0.01).

Mice treated with single agent AMD3100 or cisplatin had modest, but significant, reductions in tumor burden measured by fluorescence imaging over the course of the experiment (Fig. 6B) (p<0.05). By comparison, combination therapy with both AMD3100 and cisplatin significantly reduced total numbers of HeyA8-CXCR4-CBRN/Ar-CBC cells relative to vehicle control or either drug alone (p<0.01 and p<0.05, respectively). These differences were evident even though mice received only two weeks of treatment with these drugs. Mice implanted with HeyA8 ovarian cancer cells developed ascites, but there was no difference in overall body weights among various treatment groups (data not shown). There was a trend toward enhanced survival in mice treated with both AMD3100 and cisplatin (Fig. 6C). However, differences among AMD3100, cisplatin, and combined AMD3100 and cisplatin were not significant, although all treatments enhanced survival relative to vehicle control (p<0.05).

## Discussion

Ovarian cancer remains the leading cause of death from gynecologic malignancies with an overall five year survival of ≈50%. Poor prognosis of ovarian cancer is due in part to the fact that most patients present with advanced disease associated with intraperitoneal, liver, or systemic metastases [34]. Ovarian cancer initially is sensitive to chemotherapy with platinum-based drugs, although most patients relapse with drug-resistant tumor cells within approximately one year. These facts underscore the need for new treatment protocols and molecular targets to treat and eliminate ovarian cancer cells.

To facilitate development and optimization of CXCR4-targeted therapies in mouse models of ovarian cancer, we developed a click beetle luciferase complementation reporter for CXCR4 signaling. This reporter detects activation of CXCR4 based on recruitment of the cytosolic adapter protein, β-arrestin 2, to the ligand-bound receptor. Bioluminescence from the complementation reporter significantly increased in response to CXCL12, allowing us to use changes in light output as a quantitative measure of CXCR4 signaling and inhibition. Using this system, we measured pharmacodynamics of AMD3100 targeting CXCL12-CXCR4 in two- and three-dimensional cell-based assays and living mice with ovarian cancer. Comparing two-dimensional monolayer cultures and spheroids, we showed that inhibition of CXCL12-CXCR4 signaling with AMD3100, a clinically-approved small molecule, was less effective in three-dimensional culture. This result emphasizes known barriers to drug diffusion in spheroids and the utility of our CXCR4 signaling reporter to quantify pharmacodynamics of therapy in standard and advanced cell culture systems [35]. Two-dimensional cultures also test effects of AMD3100 to prevent activation of CXCR4 in response to acute addition of CXCL12, while spheroids quantify responses to AMD3100 in the context of ongoing, sustained CXCL12-CXCR4 signaling. Drug responses in spheroid cultures likely are more representative of treatment efficacy of AMD3100 in multicellular aggregates of ovarian cancer cells present *in vivo*.

By combining click beetle bioluminescence with whole animal fluorescence imaging, we were able to analyze changes in CXCR4 signaling relative to total tumor burden over extended periods of time in the same cohort of mice. The imaging reporter for CXCR4 activation in ovarian cancer complements our prior imaging system for directly quantifying CXCL12 binding to CXCR4 [14]. The advantage of the CXCR4-β-arrestin 2 reporter is that this system quantifies activation of CXCR4 in ovarian cancer cells by all sources of CXCL12 in the tumor microenvironment, while our previous assay detected only CXCL12 engineered with a fusion protein for complementation imaging. We readily detected bioluminescence from CXCR4-β-arrestin 2 complementation in 96 well plates (two-dimensional cultures) and 384 well plates (spheroids), suggesting the assay would be useful for high-throughput screening assays of potential CXCR4 inhibitors. The same cells then could be used to test compounds and dosing schedules in mouse models of ovarian cancer, using CXCR4-β-arrestin 2 complementation to quantify inhibition of the receptor and fluorescence to measure overall tumor burden. Collectively, our prior report and the current study establish imaging technologies to quantify key early steps in CXCL12-CXCR4 signaling and analyze targeting of therapeutic agents in pre-clinical models of ovarian cancer.

We demonstrated that combination therapy with AMD3100 targeting CXCL12-CXCR4 and the conventional drug cisplatin significantly reduced overall burden of ovarian cancer cells in the abdomen relative to treatment with either agent alone or vehicle control. These effects are consistent with prior reports in animal models of glioma and acute lymphoblastic leukemia in which responses to therapy were improved by treating mice with a standard chemotherapeutic drug and AMD3100 [36], [37]. Although we noted a trend toward extended survival in mice treated with both cisplatin and AMD3100, differences in survival relative to mice treated with cisplatin or AMD3100 alone were not statistically significant. We administered drugs for only two weeks because of challenges in managing animal health over extended periods of chemotherapy, so we treated mice for less than half of the time following injection of cancer cells. Given more sophisticated methods to administer drugs and manage side effects of therapy in humans, we anticipate that patients with ovarian cancer could be treated with an inhibitor of CXCL12-CXCR4 and cisplatin for longer periods of time. Therefore, clinical benefits of targeting CXCR4 as part of combination therapy may be greater in patients with ovarian cancer.

In addition to direct effects on ovarian cancer cells, CXCL12 and CXCR4 regulate key components of the tumor microenvironment. CXCL12-CXCR4 signaling promotes tumor angiogenesis, and blocking this signaling pathway limits vascularization of tumor metastases in mouse models of ovarian cancer [10], [38]. CXCL12 in the tumor microenvironment and ascites recruits several different types of immunoregulatory and immunosuppressive cells, including dendritic cells, T regulatory cells, and myeloid derived suppressor cells [8], [11]. These cells limit immune surveillance and control of ovarian cancer cells, contributing to disease progression. Previous studies also have reported that blocking CXCR4-dependent pathways in the tumor microenvironment is critical for therapeutic effects in cancer. In a mouse model of metastatic melanoma, genetic deletion of one copy of CXCR4 from stromal cells significantly reduced recruitment of myeloid cells to the lung and diminished overall lung metastases [39]. Studies in hematologic malignancies show that AMD3100 mobilizes malignant cells from protective niches in bone marrow, increasing susceptibility to chemotherapy [40], [41]. Similarly, blocking CXCL12-CXCR4 signaling may displace ovarian cancer cells from protective niches formed by soluble molecules and direct contact with stromal cells in the peritoneum [42]. These results suggest that treatment with inhibitors of CXCL12-CXCR4 may benefit even ovarian cancer patients in whom malignant cells do not express CXCR4.

In summary, we developed a new molecular imaging reporter based on click beetle red luciferase to quantify activation and inhibition of CXCR4 signaling in living mice. Using this imaging technology, we established therapeutic targeting of CXCL12-CXCR4 with AMD3100, a clinically-approved inhibitor of ligand receptor binding. We demonstrated that combination therapy with AMD3100 and cisplatin significantly decreased tumor growth in mice with metastatic ovarian cancer. These results suggest that incorporating an inhibitor of CXCR4 into drug protocols for ovarian cancer may improve outcomes for patients with this disease. Moreover, the imaging technology described in this report provides a facile method to assess pharmacodynamics of CXCR4 inhibitors in mouse models of ovarian cancer. We expect this imaging technique to facilitate development of new drugs targeting CXCL12-CXCR4 and streamline optimization of dosing schedules and protocols for clinical trials.
